# Emergency General Surgery: Predicting Morbidity and Mortality in the Geriatric Population

**DOI:** 10.1055/s-0042-1756461

**Published:** 2022-09-26

**Authors:** Abubaker Elamin, Panagiotis Tsoutsanis, Laith Sinan, Seyedh Paniz Hashemi Tari, Wafa Elamin, Hayato Kurihara

**Affiliations:** 1 IRCCS Humanitas Research Hospital, Rozzano, Milan, Italy; 2 Nottingham University Hospitals, Nottingham, United Kingdom; 3 Ipswich Hospital, Ipswich, United Kingdom; 4 Teesside University, Middlesbrough, United Kingdom

**Keywords:** emergency general surgery, surgery, elderly, scoring systems, APACHE II, ASA, ACS-NSQIP, clinical frailty score, Clavien–Dindo

## Abstract

**Introduction** Numerous scoring systems have been created to predict the risk of morbidity and mortality in patients undergoing emergency general surgery (EGS).

In this article, we compared the different scoring systems utilized at Humanitas Research Hospital and analyzed which one performed the best when assessing geriatric patients (>65 years of age). The scoring systems that were utilized were the APACHE II (Acute Physiology and Chronic Health Evaluation II), ASA (American Society of Anesthesiologists), ACS-NSQIP (American College of Surgeons-National Surgical Quality Improvement Program), Clinical Frailty Score, and the Clavien–Dindo classification as control.

**Materials and Methods** We compiled a database consisting of all patients over the age of 65 who underwent EGS in a consecutive 24-month period between January 1, 2017 and December 31, 2018. We used the biostatistical program “Stata Version 15” to analyze our results.

**Results** We found 213 patients who matched our inclusion criteria. Regarding death, we found that the ACS-NSQIP death calculator performed the best with an area under the curve of 0.9017 (odds ratio: 1.09; 95% confidence interval: 1.06–1.12). The APACHE II score had the lowest discriminator when predicting death. Considering short-term complications, the Clavien–Dindo classification scored highly, while both the APACHE II score and Clinical Frailty Score produced the lowest results.

**Conclusion** The results obtained from our research showed that scoring systems and classifications produced different results depending on whether they were used to predict deaths or short-term complications among geriatric patients undergoing EGS.

The phrase emergency general surgery (EGS) is defined by the American Association of Trauma as “any patient (inpatient or emergency department) requiring an emergency surgical evaluation (operative or nonoperative) for diseases within the realm of general surgery as defined by the American Board of Surgery.” 1 In the United States alone, there are approximately 130 million emergency room visits annually. 2 3 Of these, 27 million admissions are for EGS, 4 with over 30% of EGS performed being in the elderly population groups aged 65 years and older. 5

EGS encompasses a diverse number of pathologies. They are unique in that they carry an acute risk to life or long-term morbidity. Although the increased risk of morbidity and mortality has been well established, 6 the specific causative factors are poorly understood. 7 8 9 In recent decades, many scoring systems were created to help surgeon stratify the risks associated with each surgery. However, there is still no consensus on which scoring system is superior and which one to use for each surgical scenario. The five scoring systems that were used in the study will be discussed in the following sections.

## APACHE II

**Fig. 1:**
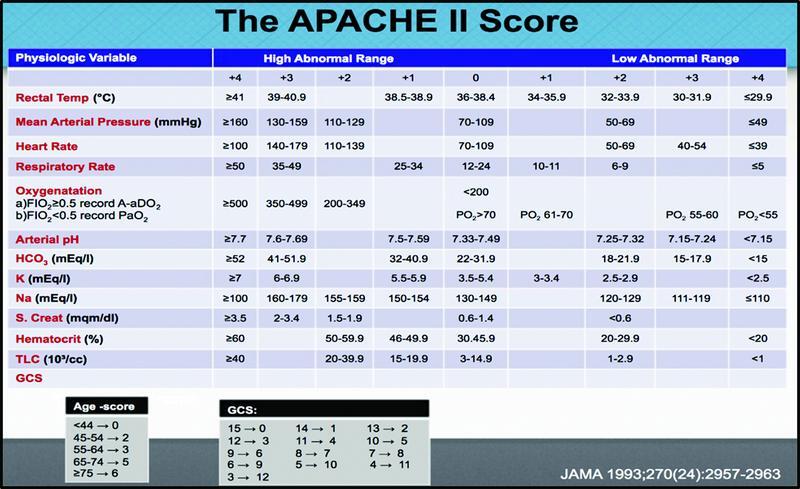
The variables included in the APACHE II score with the ranges included.

The APACHE (Acute Physiology and Chronic Health Evaluation) scoring system was first introduced by Knaus and colleagues in 1981. 10 The most frequently used model is the APACHE II ( Fig. 1 ). It is often used in the intensive care unit (ICU) to determine the severity of illness in critically unwell patients. Ideally, this scoring system should be used within 24 hours of patient admission to the hospital. In this way, it is a patient admission score. 11

## Clavien–Dindo Classification

**Fig. 2:**
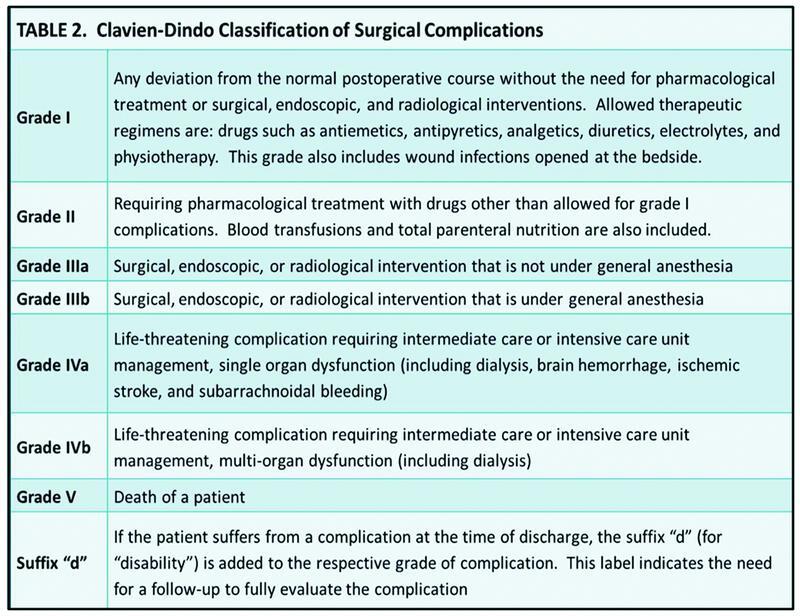
The grading system for the Clavien–Dindo classification. 14

The Clavien–Dindo classification was first proposed in 1992 and consisted of a four-level severity grading. This initial model was revised in 2004 by Dindo et al, 12 which led to the updated model utilized today ( Fig. 2 ). This system is used throughout surgery for grading and predicting adverse events or complications. 13

## American Society of Anesthesiologists Physical Classification System

**Fig. 3:**
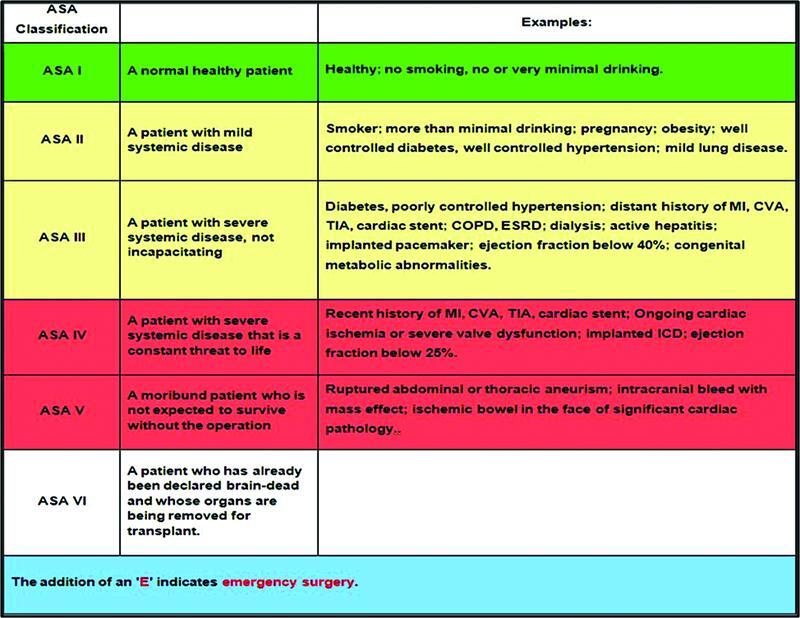
The ASA classification with each grade. 15 ASA, American Society of Anesthesiologists.

The American Society of Anesthesiologists (ASA) classification system is a subjective tool used to assess a patient's fitness to undergo surgery. It is widely employed by anesthesiologists to determine a patient's preoperative health. 14 This classification mainly assesses a patient's comorbidities ( Fig. 3 ). 15 When used alone, it is not sufficient to provide reliable information on operative risk. Therefore, it should be considered alongside other factors such as the patient's frailty level, type of surgery being performed, and the available facilities in the surgical department. One key limitation of the ASA classification system is that it is subjective and this can lead to discrepancies between different records. 16 Also, it does not consider the age and physical fitness, other comorbidities such as cancer, or the skill of the anesthesiologists or the surgeons involved.

## Clinical Frailty Score

**Fig. 4:**
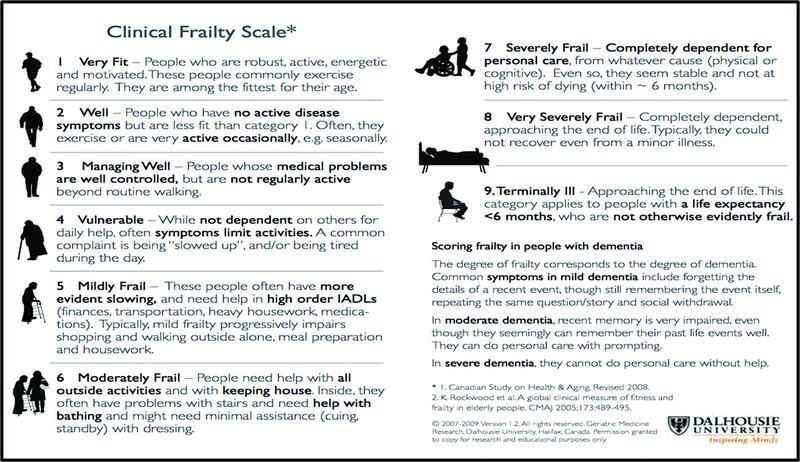
Clinical frailty scale showing all nine levels.

The Clinical Frailty Score (CFS) was introduced by the Canadian Study of Health and Aging to appropriately assess frailty. 17 18 The CFS is intended to be assessed during triage by an experienced clinician and then further reassessed after 2 weeks if appropriate ( Fig. 4 ).

The CFS has only been widely validated in patients above 65 years and should not be used in young patients or those with learning disabilities. 19 By identifying patients who are more likely to have longer hospital stays in acute units, health care professionals can help tailor patient care to prevent possible complications. 20

## ACS-NSQIP Risk Calculator

**Fig. 5:**
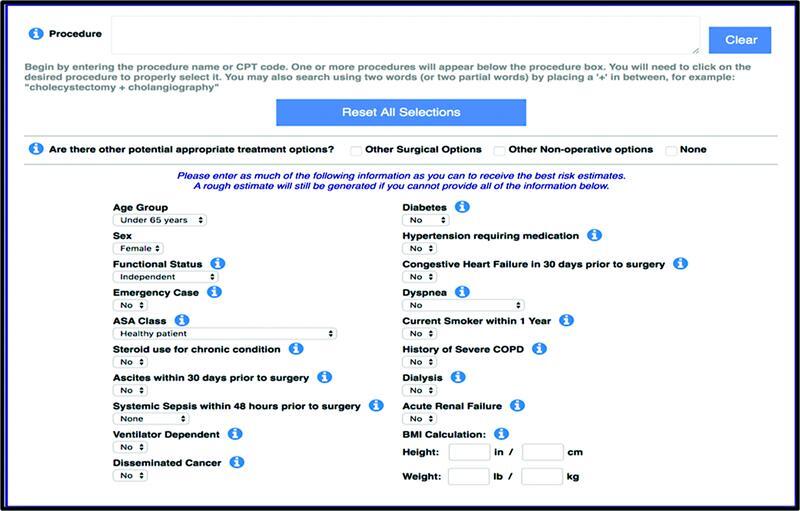
The ACS calculator with all parameters required. ACS, American College of Surgeons.

The American College of Surgeons (ACS) created the Universal Risk Calculator. Data were gathered from over 4.3 million operations performed for 3 years (2014–2017) throughout different centers in the United States. This project aimed to provide accurate and tailored patient risk information. The ACS-National Surgical Quality Improvement Program (ACS-NSQIP) Risk Calculator measures the risk of complications in the first 30 days following surgery. 21 This is important as it can help guide surgical decision making to reduce any preventable complications as well as guide the necessary postoperative care ( Fig. 5 ). 22

## Study Aim

This study aims to determine the most accurate scoring systems in predicting the risk of morbidity and mortality in geriatric patients undergoing EGS.

## Methods

A retrospective single-center cohort study was performed to assess which clinical scoring systems were better at predicting morbidity and mortality among geriatric patients. Patients were gathered from the database of all geriatric patients who underwent an EGS at Humanitas Research Hospital, Milan, Italy.

For our study we defined morbidity as any short-term complications following EGS that occurred during hospitalization. Mortality was defined as percentage of patients who died in the hospital following EGS during that admission. Our secondary end point was solely the hospital mortality.

The patient inclusion and exclusion criteria are described in Table 1 .

**Table 1:** Inclusion and exclusion criteria

Inclusion criteria	Exclusion criteria
Patients above 65 years of age Both sexes and any ethnicity	Patients ages less than 65 years
Having undergone an emergent abdominal surgery	Patients who had undergone nonacute abdominal surgery or other types of surgeries not within the abdomen
Surgery performed between 2017 and 2018	Surgery performed in any other year
Surgical pathologies: acute appendicitis, cholecystitis, diverticulitis, small bowel obstruction, peptic ulcer disease, acute mesenteric ischemia, hernias, and volvulus	Surgical pathologies not listed in the inclusion criteria

### Data Collection and Analysis

All data were anonymized and stored on secure computers within Humanitas Research Hospital computers. They were only accessible to the researchers The files were deleted from computers once data analysis was completed.

Four scoring systems were explored in the study, and these were as follows: the APACHE II, CFS, Clavien–Dindo, and ACS score. As the APACHE II is the most widely employed version of the APACHE score, it is the one that was explored throughout this study. Each patient's clinical risk was calculated according to each score or classification. These outcomes were then compared with the predicted outcomes. The statistical program used was Stata version 15.

For each patient included in our study, the respective score of each of the systems utilized was calculated postoperatively, but the data gathered were from admission results. This ensured that the achieved scores were as accurate as possible and took into account the patient preoperative state. The Clavien–Dindo scoring system takes into account postoperative complications and was used in our study as a control.

We calculated each scoring system's mean score, standard deviation, and p50 value. The odds ratio (OR) was calculated for each scoring system utilized and the associated *p-* values were used to determine statistical significance. The *p* -value was set at 0.05. The receiver operating characteristic curve was used to obtain the OR with the associated 95% confidence interval (CI). The area under the curve (AUC) was calculated to determine the level of discrimination ( Table 2 ). Initially univariate analysis was performed for each of the variables. To ensure the validity of our results further, a multivariate analysis was completed to guarantee that there were no confounding factors.

**Table 2:** AUC guidelines taken from 23

AUC	Guidelines
0.5–0.6	No discrimination
0.6–0.7	Poor discrimination
0.7–0.8	Acceptable discrimination
0.8–0.9	Good discrimination
0.9–1.0	Excellent discrimination

Abbreviation: AUC, area under the curve.

## Results

A total of 213 patients matched the inclusion criteria fully. Of these, 96 patients were aged between 65 and 74, 56 patients were aged between 75 and 80, and 60 patients were ages above the age of 80 years. A total of 116 (54.7%) of the patients were male, while the remaining 96 (45.3%) were female.

The surgeries performed included: appendectomies, cholecystectomies, enterectomies and colectomies, abdominal wall surgeries, adhesiolysis, and repair of perforations.

The overall in-hospital mortality was 16 patients, which totaled 7.55%. Postsurgical complications were accounted in 42 patients, which amounted to 19.81%. A total of 213 patients were included in our database.

The mean score, standard deviation, and p50 values were calculated for each scoring system. A summary of these findings is shown in Table 3 .

**Table 3:** Mean score, standard deviation, and p50 value for each scoring system

Variable	Mean	Standard deviation	P50	Minimum	Maximum
APACHE II	8.474178	3.876093	8	3	37
ACS serious complications	18.17371	15.15156	14.3	1.6	68.9
ACS average risk	12.11502	9.387017	10.9	1.6	32.5
ACS death	9.257746	16.30112	2.4	0	86
ACS average death	1.400469	1.835178	0.8	0.1	15.8
Frailty score	4.061033	1.971742	4	1	9

Abbreviation: ACS, American College of Surgeons.

For the ASA score and the Clavien–Dindo score, we tabulated the frequency of each grade. These can be viewed in Tables 4 and 5 .

**Table 4:** Frequency of the ASA score grades

Grade	Frequency	Percent	Cumulative
1	6	2.82	2.82
2	100	46.95	49.77
3	76	35.68	85.45
4	28	13.15	98.59
5	3	1.41	100.00
*Total*	*213*	*100*	

Abbreviation: ASA, American Society of Anesthesiologists.

**Table 5:** Frequency patient grades using the Clavien–Dindo scoring system

Grade	Frequency	Percent	Cumulative
0	83	38.97	38.97
1	39	18.31	57.28
2	46	21.60	78.87
3A	5	2.35	81.22
3B	15	7.04	88.26
4	3	1.41	89.67
4A	3	1.41	91.08
4B	2	0.94	92.02
5	17	7.98	100.00
*Total*	*213*	*100.00*	

For the ASA score, grade 2 was the most prevalent at 46.95% of all patients within this study. These patients have mild systemic disease. Patients with severe systemic disease who were not incapacitated amounted to 35.68% of this study population. Only three patients (1.41%) were categorized as grade 5, or brain dead ( Table 2 ).

For the Clavien–Dindo score, almost 39% of patients were categorized as grade 0 ( Table 3 ). This means that these patients had no risk of complications or adverse effects following the surgery and had the best overall outcomes. In addition, 18% of the patients were categorized as grade 2, meaning they had postsurgical complications that required drug treatments other than those allowed for grade I complications. This included treatments such as blood transfusions or total parenteral nutrition. Just over 21% of the patients were categorized as grade 3, meaning they had complications that required surgical, endoscopic, or radiological interventions ( Table 5 ).

The OR was calculated for all the surgical scoring systems. This was calculated for the (1) patients risk of death and (2) the short-term complications of emergency surgery. The AUC was calculated for each score, which helped determine its discrimination grade. AUC provides an aggregate measure of performance across all possible classification thresholds, which is shown for each score.

### Risk of Death

**Fig. 6:**
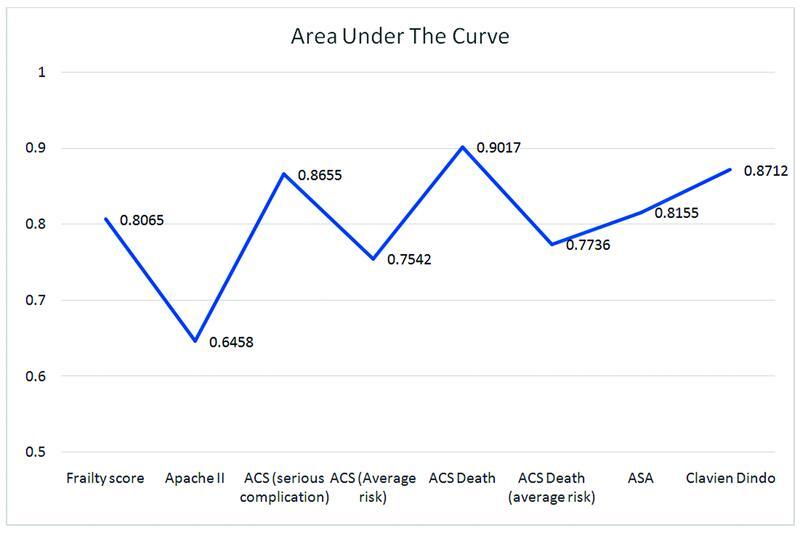
AUC of scoring systems predicting death. AUC, area under the curve.

When assessing the risk of death, all scoring systems had a positive OR (>1) ( Table 6 ). For the CFS, the OR was 1.97 with a narrow CI of 1.52 to 2.54. The AUC was 0.8065, which means that it has a high level of discrimination for the population being studied. The APACHE II score had an OR of 1.21 (95% CI: 1.10–1.34) for the risk of death. It yielded the lowest AUC with a result of 0.6458. This proved it to be a poor discriminator. The ASA score had the highest OR with a ratio of 6.48 and a wide CI (95% CI: 3.30–12.71). The AUC was 0.8155, which proved it to be a “good discriminator” for risk of death in patients undergoing emergency surgery. The Clavien–Dindo score showed “good discrimination” for death with an AUC of 0.8712 (OR: 1.99; 95% CI: 1.63–2.43). Finally, the ACS-NSQIP calculator's discriminating power was calculated by analyzing the risks of ACS serious complications and ACS death separately. When predicting death, the ACS serious complications, proved to be a “good discriminator,” with an AUC 0.8655 (OR: 1.11; 95% CI: 1.07–1.15), while the ACS death calculator was an “excellent discriminator” with an AUC of 0.9017 (OR: 1.09; 95% CI: 1.06–1.12) ( Fig. 6 ).

**Table 6:** Scoring systems for predicting death

Scoring system	Odds ratio (95% confidence interval)	AUC
Frailty score	1.97 (1.52–2.54)	0.8065
APACHE II	1.21 (1.10–1.34)	0.6458
ACS (serious complications)	1.11 (1.07–1.15)	0.8655
ACS (average risk of serious complications)	1.11 (1.06–1.17)	0.7542
ACS death	1.09 (1.06–1.12)	0.9017
ACS death (average risk)	1.65 (1.31–2.07)	0.7736
ASA	6.48 (3.30–12.71)	0.8155
Clavien–Dindo	1.99 (1.63–2.43)	0.8712

Abbreviations: AUC, area under the curve; ASA, American Society of Anesthesiologists.

### Short-Term Complications

**Fig. 7:**
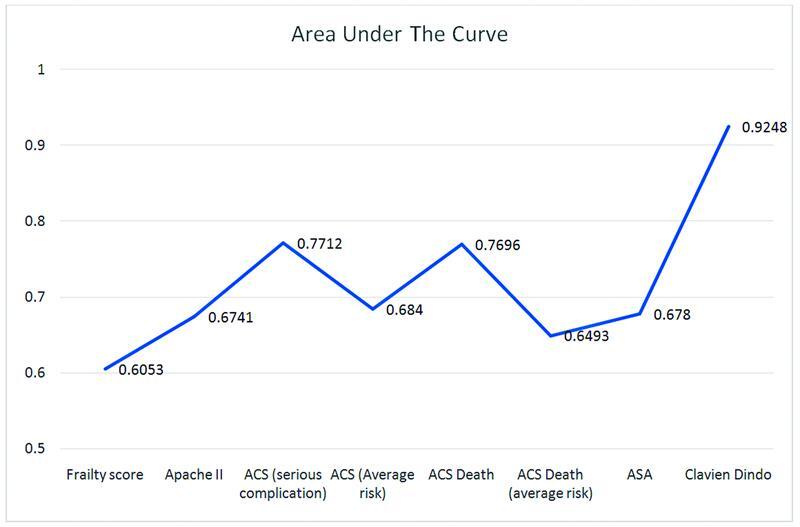
AUC of scoring systems predicting short-term complications. AUC, area under the curve.

When assessing the short-term complication of emergency surgery, all scoring systems had an OR above 1 ( Table 7 ). The scoring system with the highest AUC is the Clavien–Dindo score with a score of 0.9248, translating to a definition of “excellent discrimination” (OR: 2.05; 95% CI: 1.66–2.54). The APACHE II score for short-term complications yielded an OR of 1.17 (95% CI: 1.06–1.28). It had an AUC of 0.6741, which proved it to be a “poor discriminator.” The CFS had an OR of 1.28 (95% CI: 1.07–1.51) with an AUC of 0.6053, meaning that its degree of discrimination is classified as “poor.” The ASA score had an OR of 2.56 (95% CI: 1.60–4.10) and an AUC of only 0.6780. Again, this was classified as a “poor discriminator.” When predicting the short-term complications of emergency surgery, the ACS-NSQIP calculator was also measured. Both ACS serious complications and ACS death were “acceptable discriminators” for short-term complications with AUCs of 0.7696 (OR: 1.07; 95% CI: 1.04–1.09) and 0.7712 (OR: 1.07; 95% CI: 1.05–1.10), respectively ( Table 7 and Fig. 7 ).

**Table 7:** Scoring systems risk of short-term complication

Scoring system	Odds ratio (95% confidence interval)	AUC
Frailty score	1.28 (1.07–1.51)	0.6053
APACHE II	1.17 (1.06–1.28)	0.6741
ACS (serious complications)	1.07 (1.05–1.10)	0.7712
ACS (average risk of serious complications)	1.07 (1.03–1.11)	0.6840
ACS death	1.07 (1.04–1.09)	0.7696
ACS death (average risk)	1.24 (1.04–1.48)	0.6493
ASA	2.56 (1.60–4.10)	0.6780
Clavien–Dindo	2.05 (1.66–2.54)	0.9248

Abbreviations: AUC, area under the curve; ASA, American Society of Anesthesiologists.

## Discussion

The main objective of this article was to find which scoring system had the highest predictive power for patient morbidity and mortality when undergoing EGS. We analyzed scores that were used preoperatively and postoperatively to determine which scores had the statistically significant difference between them. Although the CFS proved to be significant in predicting mortality, similar findings were not echoed when exploring the short-term complications. This might be because the CFS is designed to assess a patient's activity level in relation to hospital stay and mortality. 18 23

According to our results, the APACHE II score was ranked as a “poor discriminator” of death as well as for short-term complications. There may be several factors that explain why the AUC score was so low. The APACHE II should be calculated within 24 hours of admission to the hospital to obtain accurate scores. 11 Unfortunately, the exact timing and condition of the patient when the score was calculated were not recorded in the data, which may explain why the AUC was low. Also, the APACHE II score is intended to be used in patients who are critically ill and admitted directly to the ICU. However, out of 213 patients, only 41 were admitted to the ICU in our study. 24 This may be the reason why the AUC was low for this measure.

In this study, the ASA score was a good discriminatory tool for predicating death; however, the score was a poor predictor of short-term complications. This is not surprising as the scoring system was initially created to predict the operative risk of adverse outcomes rather than short-term complications. 24 An intrinsic limitation of the ASA score is that it is a subjective tool. Grading may differ between anesthesiologists due to the subjective nature of the score. To obtain the most accurate results, it would be ideal to have the same doctor use this score for all the patients in the same database or study. 16 Another limitation is that this score must take into consideration other factors such as patient frailty or the facilities available.

The Clavien–Dindo score is calculated postsurgically and is dependent on the complications which occur and the treatment necessary to resolve them. For this reason, it was expected to be a reliable tool to predict patient death, which is in line with our findings. Since this score is calculated after the complications have occurred, we assumed it would have “excellent discrimination,” but instead, it fell slightly below the necessary cut-off of an AUC of 0.9. One explanation for this is that it does not consider the “failure to cure” aspect. This would mean that although the patient may not have any adverse outcomes from the surgery itself, they may die of the underlying pathology, which would not be recognized by the Clavien–Dindo scoring system. Nonetheless, the results show a consistent score with positive capabilities. Therefore, it was added to the analysis as a control to have a reliable system to compare the other scoring systems. The AUC value calculated for the Clavien–Dindo score when predicting short-term complications was the highest compared with the scoring systems, as expected. 25

Lastly, for the ACS-NSQIP calculator, both the risk of ACS serious complications and ACS death were calculated separately. Their ability to predict both short-term complications and death was explored, although the ACS-NSQIP calculator is relatively a new scoring system. 21 In terms of short-term complications, even though the ACS serious complication calculator did not perform better than the Clavien–Dindo classification, it remains the best system for predicting the likelihood of patients having a complication after EGS. The risk of “serious complications” was analyzed and doing so may have underestimated the score, as it also usually considers minor complications, which we did not include in this study.

Our study has some limitations, which included the fact that the study was performed in a single center; therefore, it is unclear whether similar results would be encountered in other hospitals. There was no follow-up of morbidity or mortality after the patients were discharged from the hospital. Finally, the analysis was not subdivided according to the patient age group, which limits us from analyzing the scoring systems used for different ages.

Multiple studies have demonstrated similar results to ours about the effectiveness of the ACS-SQIP calculator as a strong predictor of morbidity and mortality in patients undergoing EGS. 22 26 While our article analyzed the predictive ability of five common scoring systems, there are multiple other scoring systems, such as the POSSUM or completely novel approaches like using machine learning, to predict morbidity and mortality that can now be used. 27 28 29 These studies have had some positive results and it would be interesting to compare these new scoring methods to the systems that we have assessed.

## Conclusions

Many medical and surgical scoring systems are used to predict patient outcomes following general surgery. However, no studies have compared the different scores, as well as which one would be best to use. To address this issue, we decided to compare the different predicative scoring systems employed in Humanitas Research Hospital. In this study, the ACS was the most effective at predicting death, while the Clavien–Dindo classification was the best at predicting short-term complications in patients.

Further studies analyzing these different surgical scoring systems and comparing them to each other in different hospitals can help optimize patient outcomes following EGS. As the ASA score is a subjective tool, exploring patient outcomes when the same clinician assigns the score to all the study subjects may reveal interesting findings and should be explored further.
