# Addendum: Emergency General Surgery: Predicting Morbidity and Mortality in the Geriatric Population

**DOI:** 10.1055/s-0042-1758693

**Published:** 2022-11-19

**Authors:** Abubaker Elamin, Panagiotis Tsoutsanis, Laith Sinan, Seyedh Paniz Hashemi Tari, Wafa Elamin, Hayato Kurihara

**Affiliations:** 1 IRCCS Humanitas Research Hospital, Rozzano, Milan, Italy; 2 Nottingham University Hospitals, Nottingham, United Kingdom; 3 Ipswich Hospital, Ipswich, United Kingdom; 4 Teesside University, Middlesbrough, United Kingdom

 Authors of the above-mentioned article have informed that Abubaker Elamin and Panagiotis Tsoutsanis are the co-first authors. The original article was published on September 26, 2022 in issue 8.3. DOI: 10.1055/s-0042-1756461 . The article has been updated accordingly. 

